# Exosomes derived from olfactory ensheathing cells provided neuroprotection for spinal cord injury by switching the phenotype of macrophages/microglia

**DOI:** 10.1002/btm2.10287

**Published:** 2021-12-28

**Authors:** Hong Fan, Zhe Chen, Hai‐Bin Tang, Le‐Qun Shan, Zi‐Yi Chen, Xiao‐Hui Wang, Da‐Geng Huang, Shi‐Chang Liu, Xun Chen, Hao Yang, Dingjun Hao

**Affiliations:** ^1^ Department of Spine Surgery, Shaanxi Spine Medicine Research Center, Translational Medicine Center, Hong Hui Hospital Xi'an Jiaotong University Xi'an China; ^2^ Department of Neurology The Second Affiliated Hospital of Xi'an Jiaotong University Xi'an China; ^3^ Department of Laboratory Medicine, Xi'an Central Hospital Xi'an Jiaotong University Xi'an China; ^4^ Department of Endocrinology The First Affiliated Hospital of Xi'an Jiaotong University Xi'an China; ^5^ Department of Bone Microsurgery, Hong Hui Hospital Xi'an Jiaotong University Xi'an China

**Keywords:** immunotherapies, nanobiology, regenerative medicine

## Abstract

Transplantation of olfactory ensheathing cells (OECs) has been demonstrated to be beneficial for spinal cord injury (SCI) by modulating neuroinflammation, supporting neuronal survival and promoting angiogenesis. Besides OECs, the conditioned medium (CM) from OECs has also been proved to have therapeutic effects for SCI, indicating that the bioactive substances secreted by OECs are essential for its protective effects. Nevertheless, there is still little information regarding the underlying mechanisms. Considering that exosomes are crucial for intercellular communication and could be secreted by different types of cells, we speculated that the therapeutic potential of OECs for SCI might be partially based on their exosomes. To examine whether OECs could secret exosomes, we isolated exosomes by polyethylene glycol‐based method, and identified them by electron microscopy study, nanoparticle tracking analysis (NTA) and western blotting. In view of phagocytic ability of microglia and its distinct roles in microenvironment regulation after SCI, we then focused the effects of OECs‐derived exosomes (OECs‐Exo) on microglial phenotypic regulation. We found that the extracted OECs‐Exo could be engulfed by microglia and partially reverse the LPS‐induced pro‐inflammatory polarization through inhibiting NF‐κB and c‐Jun signaling pathways in vitro. Furthermore, OECs‐Exo were found to inhibit the polarization of pro‐inflammatory macrophages/microglia while increased the numbers of anti‐inflammatory cells after SCI. Considering that the neuronal injury is closely related to the activation state of macrophages/microglia, co‐culture of microglia and neurons were performed. Neuronal death induced by LPS‐treated microglia could be significantly alleviated when microglia treated by LPS plus OECs‐Exo in vitro. After SCI, NeuN‐immunostaining and axonal tract‐tracing were performed to assess neuronal survival and axon preservation. Our data showed that the OECs‐Exo promoted the neuronal survival and axon preservation, and facilitated functional recovery after SCI. Our findings provide a promising therapeutic strategy for SCI based on exosome‐immunomodulation.

## 1 INTRODUCTION

Spinal cord injury (SCI) often resulted in variable degrees of irreversible and lifelong neurological dysfunction.^1^, ^2^ In the past decades, efforts in searching an effective approach for SCI never stopped, and cell‐based therapy was applied to SCI.^3^, ^4^, ^5^, ^6^ Multiple types of cells have been investigated as potential therapies for SCI, including stem cells or progenitor cells, Schwann cells, olfactory ensheathing cells (OECs), which are reaching early‐phase human clinical trials.^4^, ^7^ The neurological dysfunction after SCI was mostly attributed to the disruption of the ascending and descending tracts,^8^ cells capable of inducing axon regeneration was promising for functional reconstruction of SCI.^7^ OECs have been demonstrated to have multiple beneficial effects on SCI, such as enhancing axonal regeneration, modulating neuroinflammation, supporting neuronal survival and promoting angiogenesis.^8^, ^9^, ^10^, ^11^ Besides transplantation of OECs, it has been proved that only delivery of conditioned medium (CM) of OECs could promote the functional recovery and axonal regeneration after contusive SCI, indicating the bioactive substances secreted by OECs are crucial for the protective effects.^12^ However, the underlying mechanism remains unclear.

Exosome, a class of extracellular vesicles released by most types of cells, served as important intercellular messengers through bioactive substances contained, such as protein, lipid, and nucleic acid.^13^, ^14^, ^15^, ^16^ Whether that OECs could secrete exosomes and the OECs‐derived exosomes (OECs‐Exo) partially mediated the therapeutic potential of OECs is worthy of further investigation. Based on our pilot study that about 50% of the OEC‐Exo was taken up by microglia/macrophages around the lesion after SCI, with less than 5% of OEC‐Exo engulfed by neurons, we speculated that OEC‐Exo might mainly play a beneficial effect for SCI by indirectly providing a favorable environment. Considering that the diverse immune microenvironment mediated by distinct phenotypic microglia/macrophages correlated with pathological outcome of SCI,^17^, ^18^, ^19^, ^20^ the present study mainly focused on exploring the effects and mechanisms of OECs‐ Exo on microglia/macropahges polarization, as well as its neuroprotective effects.

We first extracted and identified exosomes from purified OEC supernatant by polyethylene glycol‐based method,^21^ electron microscopy study, nanoparticle tracking analysis (NTA), and western blotting. We further demonstrated exosomes could be engulfed by primary cultured microglia and partially reversed the LPS‐induced M1 polarization through NF‐κB and c‐Jun signaling inhibition in vitro. After SCI, OECs‐Exo could also inhibit the polarization of pro‐inflammatory macrophages/microglia while increased the numbers of anti‐inflammatory cells. Our data further demonstrated that the OECs‐Exo promoted the neuronal survival, increased preservation of axons, and facilitated functional recovery after SCI. Thus, our findings provide a promising therapeutic strategy‐based on exosome‐immunomodulation for SCI treatment.

## 2 MATERIALS AND METHODS

### 2.1 Experimental design

A total of 120 male Sprague Dawley rats (about 200 g) were bought from the Animal Center of Xi'an Jiaotong University. Animal Care and Use Committee of Xi'an Jiaotong University approved our experimental protocols of the animals. These SD rats were divided randomly into three groups as follows: (i) in sham control group received laminectomy only; (ii) in the SCI + PBS group, animals were subjected to SCI surgery and received the same volume PBS as OEC‐Exo; (iii) in the SCI + OEC‐Exo group, animals were subjected to SCI surgery and three injections of 0.5 μl of OEC‐Exo (200 μg/ml), with one injection in epicenter and two injections 0.5 mm bilaterally from lesion immediately after SCI.

### 2.2 SCI model

The SCI surgery was established as we describe previously.^19^, ^22^ In brief, rats were anesthetized with 1% sodium pentobarbital (60 mg/kg), followed by laminectomy of vertebrae T8. The exposure spinal cord was crushed by the forceps (53327T, 66 Vision‐Tech Co., Ltd.) with 0.5 mm‐width when closed for 20 s. Then, PBS or OEC‐Exo was transplanted into the crush lesion.

### 2.3 Axonal tract‐tracing

Various adeno associated viral vectors (AAV) 2/5 green fluorescent protein (GFP, OBiO Technology, 3.06 × 10^13 gc/ml) was injected into two segments rostral to lesion of T8 three days after injury. AAV 2/5‐GFP was injected into two sites (one on each side of the cord) × 0.25 μl 1.1 mm below the surface at 0.05 μl/min using glass micropipettes connected via high pressure tubing (RWD Life Science) to 10‐μl syringes under control of microinfusion pumps.^23^ Spinal cords were harvested for evaluation of axons preservation at 4 weeks after SCI.

### 2.4 Culture and characterization of primary OECs

Primary OECs were cultured from rat olfactory bulbs and purified as described previously.^24^, ^25^, ^26^ The olfactory bulbs were obtained with Hank's 1× (Sigma) solution and digested with collagenase 0.1% (Sigma) for 10 min. The digestion was terminated and the cell pellet was suspended in DF‐12 medium (Gibco) containing 20% fetal bovine serum (FBS; Gibco), penicillin 100 IU/ml (Sigma), and streptomycin 100 mg/ml (Sigma). The method based on the differing rates of attachment of different cells was applied and the purified cell suspension was reseeded on 75‐cm^2^ poly‐lysine‐pretreated culture flasks for supernatant and on poly‐lysine‐pretreated slide for fluorescent staining. The OECs were identified by P75NTR antibody (ab52987, 1:500; Abcam).

### 2.5 Preparation and identification of exosomes from OECs

To prepare exosomes, particle free culture medium was added to OECs and collected after 48 h. Exosomes were extracted by polyethylene glycol‐based method according to other and our previous studies.^21^, ^27^ Briefly, for eliminating cell debris, the culture medium was obtained and continuously centrifuged at 500*g* for 5 min and 3000*g* for 30 min at 4°C. The supernatants were then filtrated through a 0.22‐μm filter (Millipore), and mixed with polyethylene glycol 6000 (PEG6000) (Sigma) solution to reach the final concentration of 12% PEG6000. After incubated at 4°C for more than 12 h, the mixtures were centrifuged at 12,000*g* for 1 h at 4°C. The supernatants were removed carefully, and the pellets were resuspended in PBS. The ZETASIZER Nano series‐Nano‐ZS were applied to test the size of exosomes.

The morphology of exosomes was assessed by transmission electron microscopy (TEM, JEM‐1230; JEOL Ltd.). After fixed in 3% (wt/vol) glutaraldehyde and 2% paraformaldehyde (PFA)‐cacodylate buffer, exosome pellets were loaded to copper grids coated with Formvar. Following washing buffer and distilled water washes, the grids were contrasted in 2% uranyl acetate, dried, and then examined by TEM.

To further confirm the extracted exosomes, they were identified by western blotting with biomarkers of CD9 and CD63, which were regarded as the common markers of exosomes.^28^, ^29^, ^30^

### 2.6 Exosomes uptake by microglia in vitro

OECs‐derived exosomes (OEC‐Exo) were labeled with DiI (Invitrogen) to detect the uptake of exosomes by microglia in vitro. Exosomes in PBS were mixed with DiI at a concentration of 20 μM and incubated at 37°C for 20 min. The stained exosomes were centrifuged at 12,000*g* for 30 min at 4°C and then washed with PBS for three times. After staining, exosomes were washed with PBS to remove unbound DiI. The pellets were resuspended and added to cultured microglia at indicated time points. The uptake of DiI‐labeled OEC‐Exo by microglia was observed under brightfield microscope or confocal microscope after immunostaining with anti‐Iba1 antibody (019‐19741, 1:1000; Wako). The images were captured using a confocal microscope (FV1000; Olympus) and analyzed by the ImageJ software.

### 2.7 Microglial cell culture and treatment

Rats at postnatal day 3 were adopted for microglial cells as described previously.^19^ Microglia from spinal cord were cultured in DMEM with 3% FBS, penicillin–streptomycin (100 U/ml), and 4 mM L‐Glutamine. For M1 polarization, 100 ng/ml of LPS was added into culture for 24 h. For OEC‐Exo treatment, OEC‐Exo were added into culture medium with final concentration of 5, 10, and 20 μg/ml, respectively. At the end of treatment, total RNAs were collected for quantitative real‐time polymerase chain reaction (qRT‐PCR), total proteins were obtained for western‐blotting, and cells were collected for immunostaining.

### 2.8 Neuron culture

Rat spinal cord neurons were obtained from E13‐15 rat embryos as we previously described.^27^ Primary neurons were cultured in the lower 12‐well plates (Corning). Cells were cultured in Neurobasal medium (Invitrogen) containing B27 supplement 2 mM L‐Glutamine and antibiotics. Medium was half changed every other day. After 7 days culture, neurons were cocultured with M1microglia with/without OECs‐Exo.

### 2.9 Coculture system

To observe the effect of phenotypic changes of microglia on neurons, primary neurons and primary microglia were organized in a transwell system. Primary neurons were cultured in the lower 12‐well plates (Corning). For the microglia–neuron coculture experiments, primary microglia were first seeded onto transwell filters (0.4 μm pore; Corning), and then treated with 100 ng/ml of LPS or LPS plus 20 μg/ml of OEC‐Exo for 24 h. After medium changed, microglia were added to spinal cord neurons in Neurobasal medium. After cultured in the system for 24 h, neurons in 12‐well plates were prepared for flow cytometry.

### 2.10 Flow cytometry

The apoptosis assay was performed by the PE Annexin V Apoptosis Detection Kit I (BD Pharmingen) utilizing Canto II (BD) as instructed. Briefly, neurons in normal condition, co‐cultured with M1 microglia (M1) or OEC‐Exo‐treated M1 microglia (M1 + OEC‐Exo) were collected and washed twice with cold PBS. After neurons suspended at a concentration of 10^6^ cells/ml, 100 μl of the cell suspension was pipetted, and 5 μl of PE Annexin V and 7‐AAD were added. After incubating for 15 min at room temperature in the dark, 400 μl 1× binding buffer was added in each tube and the samples were analyzed by flow cytometry within 1 h. The results were analyzed using FlowJo software.

### 2.11 Quantitative real‐time polymerase chain reaction

Total RNA was extracted from 1.0 cm length of injured cord segments or cultured cell via TRizol (Takara). Then extracted RNA received reverse transcription utilizing a reverse transcript kit (Takara). We performed real‐time PCR for TNFα, iNOS, CD86, IL‐12, IL‐18, CD206, Arginase1, IL‐4, IL‐10, and IRF‐3 with SYBR green (Takara) on Bio‐Rad PCR system (Bio‐Rad). The primers of these genes are listed in Table S1. Gene expression levels were determined by 2^−ΔΔCT^ method and the data were presented normalized by GAPDH.

### 2.12 Western blot analysis

Injured cord segments around the lesion epicenter with 0.5 cm length were obtained to detect the expression of NF200, while cells were harvested at the designed time points. Total protein was extracted with ice‐cold radioimmunoprecipitation assay (Beyotime Biotechnology) buffer containing 1% phenylmethylsulfonyl fluoride (Beyotime Biotechnology). Proteins were separated on 10% sodium dodecyl sulfate–polyacrylamide gel and electro‐transferred to polyvinylidene fluoride (Milipore) membrane. The membranes were incubated with primary antibodies against CD9 (ab 92726, 1:1500; Abcam), CD63 (gtx17441, 1:1000; Genetex), p‐NF‐κB (3033t, 1:2000; Cell Signaling Technology), p‐c‐Jun (3270s, 1:2000; Cell Signaling Technology), NF200 (1:500, ab134306; Abcam), β‐actin (A5441, 1:3000; Sigma), GAPDH (10494, 1:4000; Proteintech) at 4°C overnight. After washed with Tris‐buffered saline Tween 20, the membranes were incubated with appropriate horseradish peroxidase‐conjugated secondary antibodies at RT for 1 h. The bands were detected by enhanced chemiluminescence (Milipore) and visualized using a Bio‐Rad Image Lab system.

### 2.13 Tissue processing

At the end of the designed time points, rats were anesthetized and perfused with 200 ml 0.01 M PBS and 350 ml 4% paraformaldehyde (PFA). Segments of 20 mm were taken with the injured site as the center, and dehydrated in 30% sucrose for 48 h at 4°C.

The samples were embedded into optimal cutting temperature compound (Sakura), and both serial 10‐μm‐thick transverse sections and serial 12‐μm‐thick sagittal sections were cut on a cryostat. The sections were stored for immunostaining.

### 2.14 Immunofluorescence

For immunocytochemistry, OECs and microglia were washed with PBS, and fixed in 4% PFA for 20 min at room temperature. After blocked with 3% BSA for 15 min, cells were stained with primary antibodies: P75NTR (ab52987, 1:500; Abcam), iNOS (ab49999, 1:200; Abcam), F4/80 (SAB5500103, 1:100; Sigma), p‐c‐Jun (3270s, 1:5000; Cell Signaling Technology), and p‐NF‐κB (3033t, 1:2000; Cell Signaling Technology) at 4°C overnight. The cells were then stained with corresponding secondary antibodies. A confocal microscope (LSM 800; Zeiss) was utilized for examination and photography.

For immunohistochemistry, slides of injured spinal segments were blocked in 0.01 M PBS containing 3% BSA and 0.1% Triton X‐100 for 20 min. Subsequently, slides were incubated with primary antibodies: Iba1 (019‐19741, 1:1000; Wako), iNOS (ab49999, 1:200; Abcam), Arginase1 (sc‐166920, 1:200; Santa Cruz Biotechnology), GFAP (1:2000; Sigma), NeuN (#24307, 1:200; Cell Signaling Technology) at RT overnight. The slides were then stained with corresponding secondary antibodies, and counterstained with Hoechst 33342 to display nuclei. Slides were photographed by a confocal microscope (LSM 800; Zeiss).

### 2.15 Basso, Beattie, and Bresnahan (BBB) locomotor rating scale

BBB scores was used for assessment of locomotor recovery after SCI.^31^, ^32^ Rats were tested at 1 day before and 1, 3, 7, 10, 14, 21, and 28 days after SCI. BBB scores ranges from 0 to 21 points, with evaluation of behaviors involving the ankle, hip, trunk, tail, and hindlimb. The scores were recorded by independent investigators blinded to the treatment.

### 2.16 Rump‐height index (RHI) assay

The RHI was applied to estimate of the ability to support body weight. The movements of rats were photographed when they were walking through the left to right side on a runway bar at the same points as BBB scoring. The RHI is defined as the height of the rump, normalized to the thickness of the beam, measured along the same vertical line. To minimize the variations of pre‐surgery RHI of each rat, we modified the RHI as standardized RHI (dividing post‐injury value by pre‐injury value) as we used previously.^22^

### 2.17 Quantification

In the present study, for quantification of cells of in vitro experiments, pictures of eight random fields were obtained. All of the cells were counted and quantified by Image Tool. For quantification of cells in tissue, the profile counting followed by empirical method was applied to gain unbiased data as described.^33^, ^34^ All the positive stained cells in defined area in all sections from the randomly chosen slide were counted, followed by calibration with the empirical method, as in our recent work.^19^ Independent counting was performed by another investigator. For axon quantification, the AAV2/5‐GFP labeled axons at 2 mm rostral to the lesion, in epicenter and 2 mm caudal to the lesion was recorded in all sections,^35^ and quantified by observers blind to experimental grouping.

### 2.18 Statistical analysis

Statistical analyses were performed using Graph Pad Prism 8.0. For two group comparisons, significance was assessed by Student's *t*‐test, and one‐way analysis of variance followed by post hoc Tukey's analysis was applied for multiple group comparisons. All data were presented as mean ± standard deviation (*SEM*). Values of *p* < 0.05 were considered statistically significant.

## 3 RESULTS

### 3.1 Characterization and identification of OECs

**FIGURE 1:**
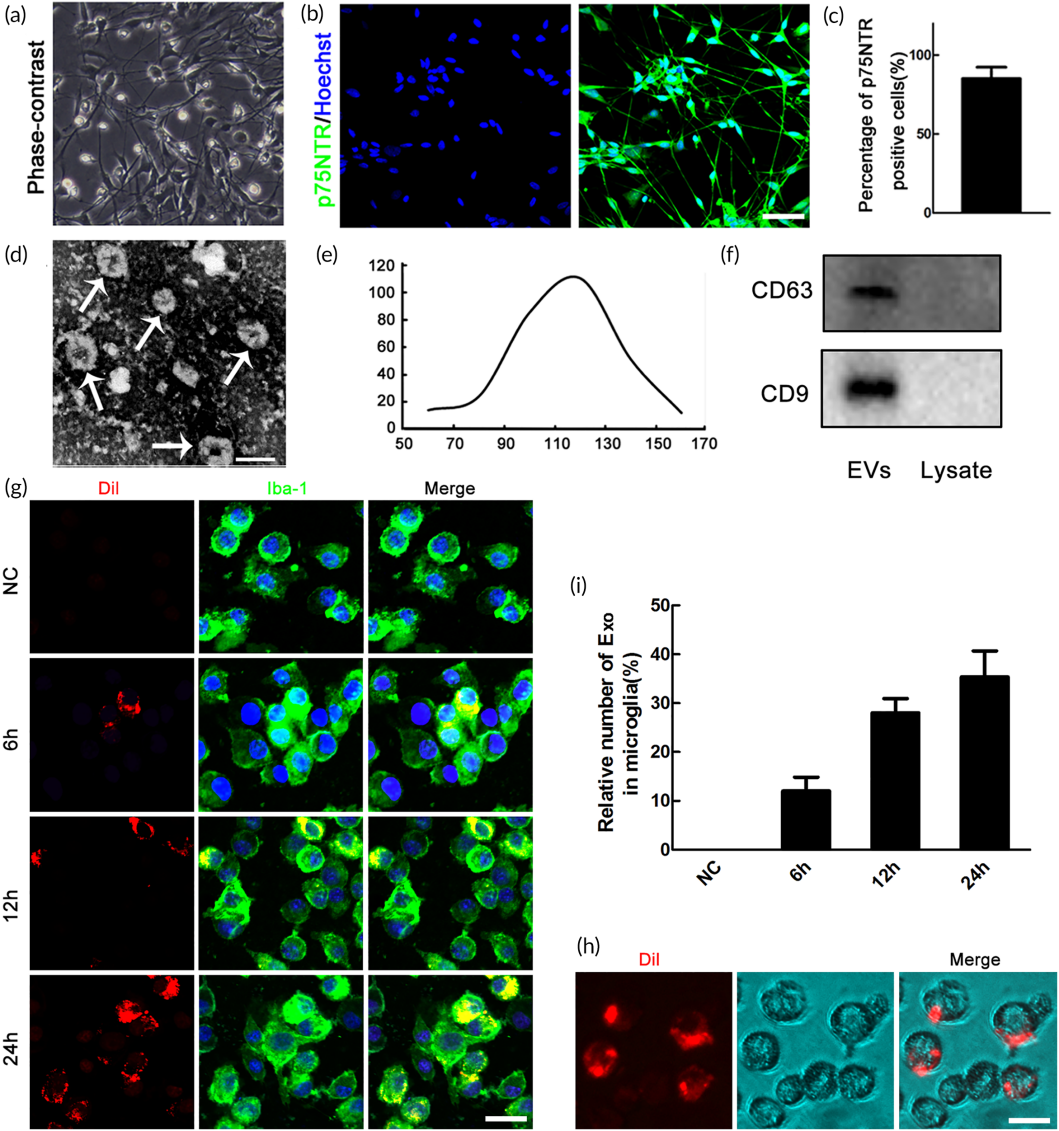
Characterization of OECs‐derived extracellular vesicles (EVs) and uptake of DiI‐labeded OECs‐Exo by microglia. (a) Identification of primary cultured olfactory ensheathing cells (OECs). Brightfield photomicrographs of olfactory ensheathing cells. (b) Immunocytochemistry of P75NTR. (c) Statistics revealed that about 90% of the purified cells were p75NTR positive. Scale bar = 50 μm. (d) Representative transmission electron microscope (TEM) image showing the morphology of OECs‐EVs. Scale bar = 120 nm. (e) Nanoparticle tracking analysis (NTA) showing the size distribution of OECs‐EVs. (f) Western blotting analysis showing the presence of EVs marker proteins of CD9, CD63 in OECs‐EVs. (g) Representative confocal microscopy images showing time‐dependent uptake of DiI‐labeded OECs‐Exo (red) by microglia (green). Note that no OECs‐Exo in negative control (NC) groups. (h) Representative images of brightfield microscopy representing DiI‐labeded OECs‐Exo (red) in microglia, and the corresponding quantification (i) of changes of relative fluorescence intensity at different time points. Scale bar = 10 μm

Primary OECs were cultured, and the representative morphology of purified OECs was first examined by phase‐contrast microscopy (Figure 1a). Immunostaining of p75NTR was further performed to identify the purified OECs, and the data showed that about 90% of the purified cells were p75NTR positive (Figure 1b,c). The exosomes used in this study were obtained from the purified OECs.

### 3.2 Isolation and identification of exosomes from OECs

To define whether OECs could secrete extracellular vesicles (EVs), we tried to isolate and identify EVs from purified OECs. The morphology was studied by transmission electron microscope (TEM), and the representative micrograph showed that the EVs have quasi‐circular membrane (Figure 1d). Nanoparticle tracking analysis (NTA) was performed to analyze the size distribution of secreted particles, and the data showed that most particles ranged from 50 to 150 nm in diameter (Figure 1e). Meanwhile, common exosome markers, including CD63 and CD9 were also confirmed by western blotting, and the results showed that these markers were enriched in EVs instead of cell lysate (Figure 1f). The above data indicated that the EVs we isolated hold the characteristics of exosomes, and these extracted exosomes (OECs‐Exo) were used in the following study.

### 3.3 Uptake of DiI‐labeled OECs‐Exo by microglia

Considering that microglia/macrophages are the major professional phagocytes after SCI, we investigated that whether OECs‐Exo could be up taken by microglia. The DiI‐labeled OECs‐Exo were added to cultured microglia at different time points. Internalization of OECs‐Exo were verified by confocal and bright‐field microscopy, and the data showed that OECs‐Exo could be engulfed by microglia in a time‐dependent manner (Figure 1g–i).

### 3.4 OECs‐Exo partially reversed M1 polarization of microglia through inhibition of NF‐κB and c‐Jun in vitro

**FIGURE 2:**
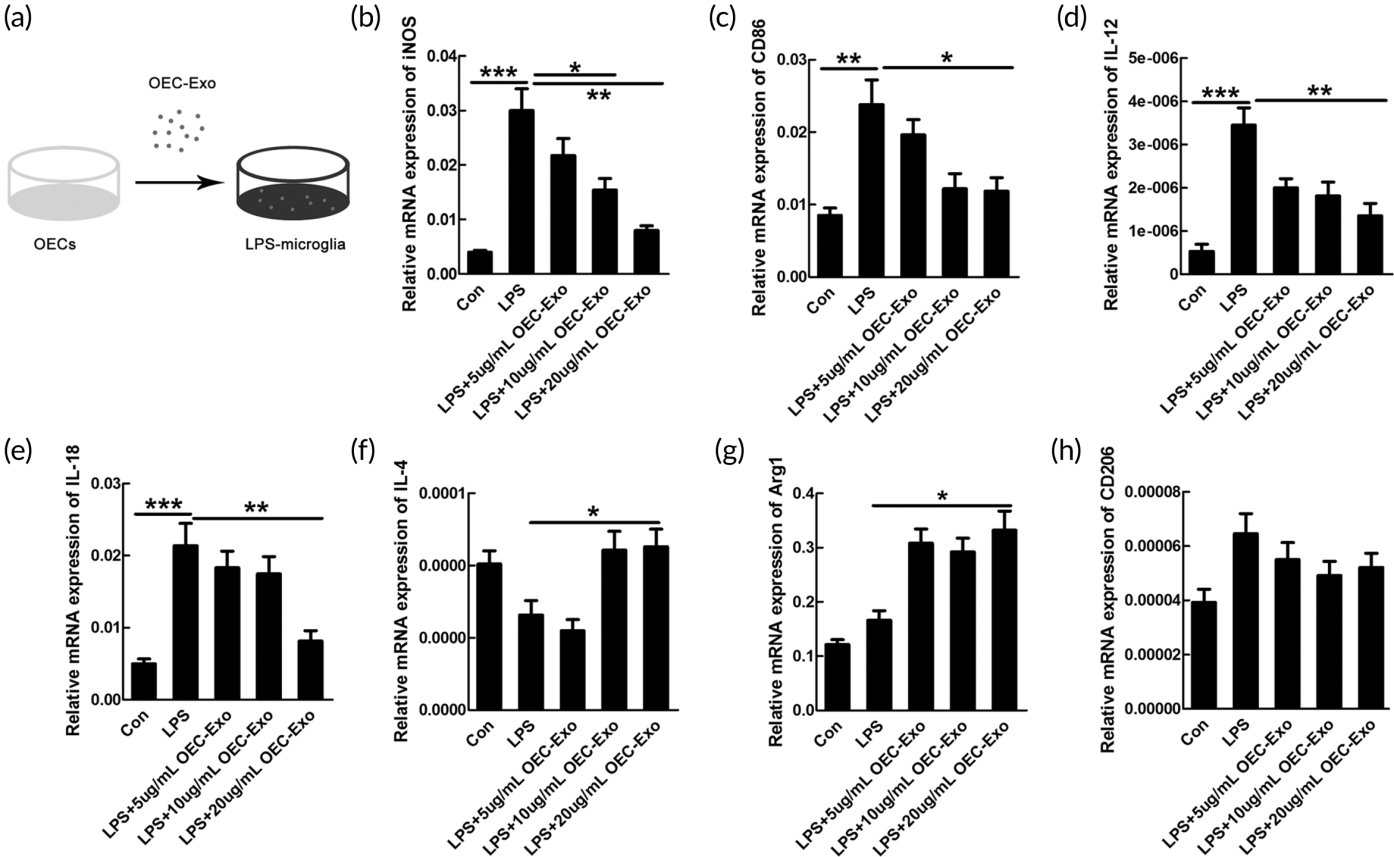
OECs‐Exo reversed pro‐inflammatory markers in microglia induced by LPS treatment. (a) Schematic diagram showing adding OECs‐Exo into microglia pretreated with LPS. OECs‐Exo could reverse the induced mRNA expression of pro‐inflammatory markers of iNOS (b), CD86 (c), IL‐12 (d), and IL‐18 (e) in microglia upon LPS treatment in a concentration‐dependent manner. OECs‐Exo could increase the mRNA levels of anti‐inflammatory markers of IL‐4 (f) and Arg1 (g), except for no significant change of CD206 (h). Results are presented as mean ± *SEM*. *N* = 3, *p < 0.05, **p < 0.01, ***p < 0.001

**FIGURE 3:**
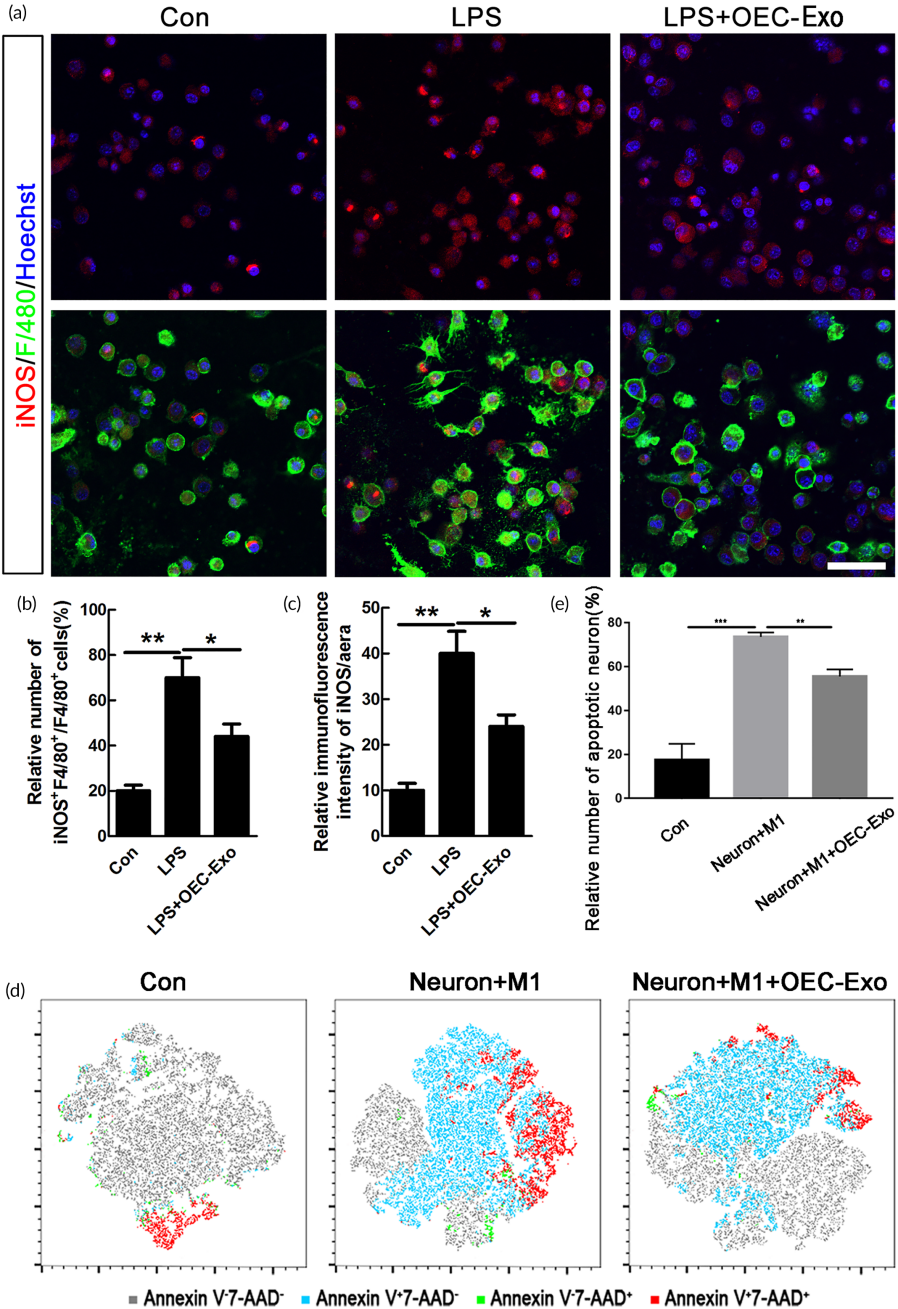
OECs‐Exo inhibited LPS‐induced pro‐inflammatory polarization of microglia. (a) Representative images of staining of iNOS and F4/80 in microglia under normal condition, LPS or LPS plus OECs‐Exo treatment. Scale bar = 30 μm. (b,c) Quantification of the numbers of iNOS‐positive cells and the IFI/area of iNOS. Note that OECs‐Exo significantly inhibited the increased number of iNOS‐positive cells and IFI of iNOS induced by LPS. *N* = 3, *p < 0.05, **p < 0.01. IFI, immunofluorescence intensity. (d) Neurons were stained with PE Annexin V and 7‐AAD in blank, co‐culture with M1 microglia, M1 microglia treated with OEC‐Exo respectively. The representative plots from flow cytometry were shown as the indicated by the T‐distributed random neighbor embedding (tSNE) format. The gray, blue, green, and red dots denoted Annexin V‐7‐AAD‐, Annexin V + 7‐AAD‐, Annexin V‐7‐AAD+, Annexin V + 7‐AAD+ subsets respectively. (e) Quantification of the relative number of apoptotic neurons (Annexin V +). *N* = 3, ***p < 0.001, **p < 0.01

Considering that M1‐polarized microglia/macrophages dominated the process of the secondary injury of SCI, emerging strategies were aimed to regulate their phenotype. Purity of microglia was first identified by Iba1‐immunostaining, and the data showed that about 90% of the cells were Iba1 positive (Figure S1). To elucidate whether efferocytosis of OECs‐Exo influences the M1‐polarization of microglia, we added the OECs‐Exo with concentration of 5, 10, and 20 μg/ml into the LPS‐treated microglia (Figure 2a). We then examined the mRNA levels of pro‐inflammatory or anti‐inflammatory phenotypic markers in control, LPS‐treated, LPS + OECs‐Exo (with different concentration) treated microglia in vitro. The data showed that OEC‐Exo treatment significantly decreased the mRNA levels of pro‐inflammatory markers of iNOS, CD86, IL‐12, and IL‐18 in LPS‐induced M1 microglia in a concentration‐dependent manner (*n* = 3, ***p < 0.001, **p < 0.01, *p < 0.05, Figure 2b–e). Meanwhile, the mRNA levels of anti‐inflammatory markers of Arg‐1 and IL‐4 were significantly increased in M1‐polarized microglia upon OEC‐Exo treatment (*n* = 3, *p < 0.05, Figure 2f,g), with no significant change of another anti‐inflammatory marker of CD206 (Figure 2h). As 20 μg/ml of OECs‐Exo exerted the strongest effect on inhibition of M1 polarization, this concentration was adopted for further experiments. Immunostaining was further performed to confirm the data of qPCR, and the data showed that OEC‐Exo significantly reduced the enhanced numbers of iNOS positive cells and the increased immunofluorescence intensity (IFI) of iNOS upon LPS treatment (*n* = 3, **p < 0.01, *p < 0.05, Figure 3a–c). Coculture of microglia–neurons was further performed to confirm the polarization of microglia. By flow cytometry, we found that M1 microglia could induce neuronal apoptosis, and this neurotoxic effect could be significantly alleviated when co‐culture with M1 microglia treated by OECs‐Exo (*n* = 3, ***p < 0.001, **p < 0.01, Figure 3d,e). The above data indicated that OEC‐Exo could switch microglia from pro‐inflammatory phenotype to anti‐inflammatory state in vitro.

We further explored the possible mechanism of the above effect by examining the interferon regulatory factor 3 (IRF‐3), c‐Jun, and NF‐κB signaling pathways, which were reported to be participated in transcriptional regulation of M1 polarization of macrophages/microglia.^36^, ^37^, ^38^, ^39^ As shown in Figure S2a, the mRNA level of IRF‐3 showed no significant changes in LPS + OEC‐Exo treated microglia, compared to LPS‐treated group (Figure S2a). The expressions of NF‐κB and c‐Jun were detected by western blotting, and our results demonstrated that OEC‐Exo could significantly decreased the induced expression of NF‐κB and c‐Jun upon LPS treatment (*n* = 3, **p < 0.01, *p < 0.05, Figures S2b and 4a,b). The immunostaining data further revealed that the increased IFI and nuclear expression of NF‐κB and c‐Jun was decreased when OEC‐Exo was added to M1 microglia (*n* = 3, ***p < 0.001, **p < 0.01, *p < 0.05, Figures S2c–e and 4c–e).

**FIGURE 4:**
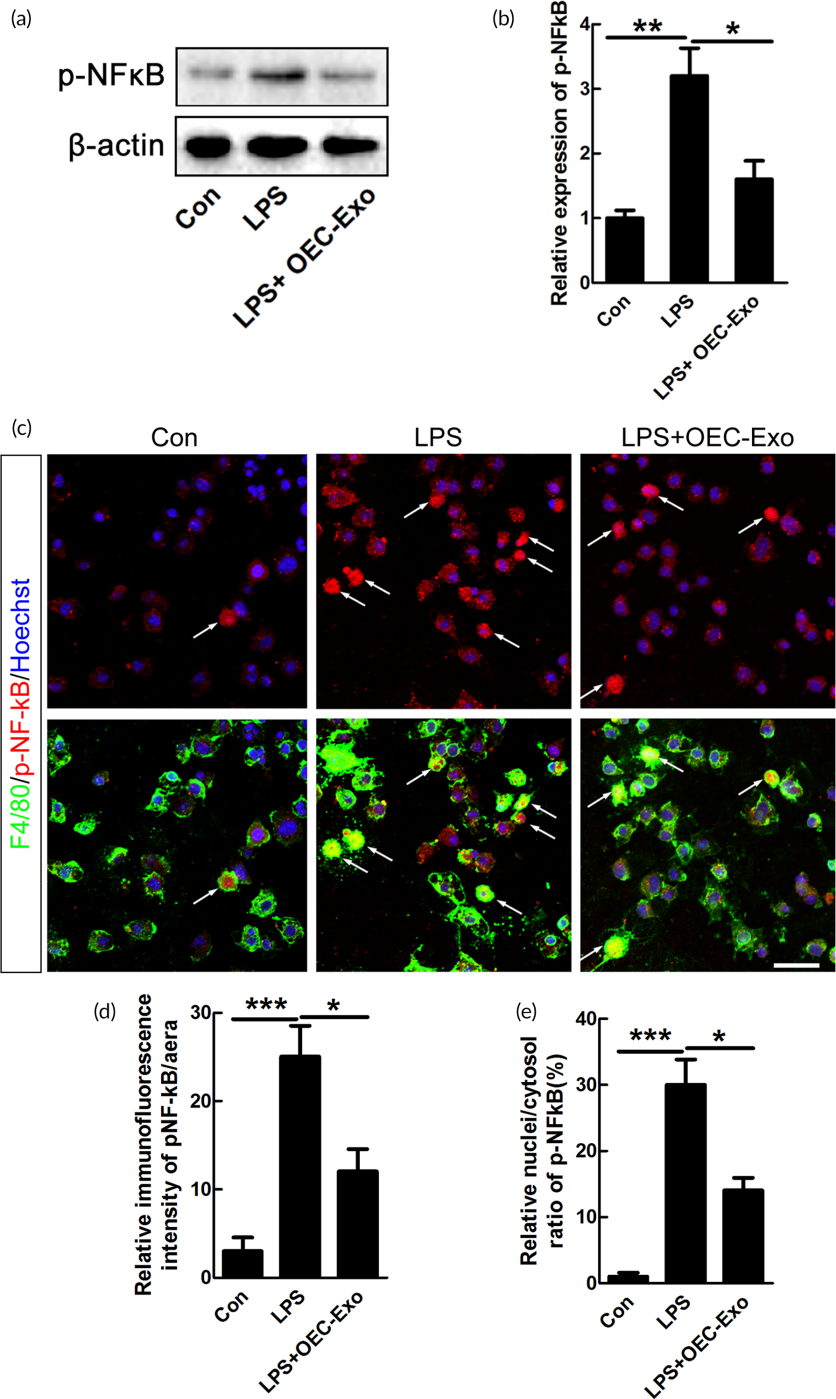
Effects of OECs‐Exo on expression of pNF‐κB after LPS treatment. (a) Expression of pNF‐κB in microglia under control, LPS or LPS plus OECs‐Exo treatment. (b) Quantification of expression level of pNF‐κB normalized to β‐actin. *N* = 3, *p < 0.05, **p < 0.01. (c) Immunostaining of pNF‐κB and F4/80. Scale bar = 30 μm. (d,e) Quantification of the IFI/area and the nuclei/cytosol fluorescence intensity ratio of pNF‐κB. Note that OECs‐Exo significantly suppressed the increased IFI and nuclear translocation of pNF‐κB induced by LPS. *N* = 3, *p < 0.05, ***p < 0.001

### 3.5 OEC‐Exo regulated the polarization of macrophage/microglia after SCI

**FIGURE 5:**
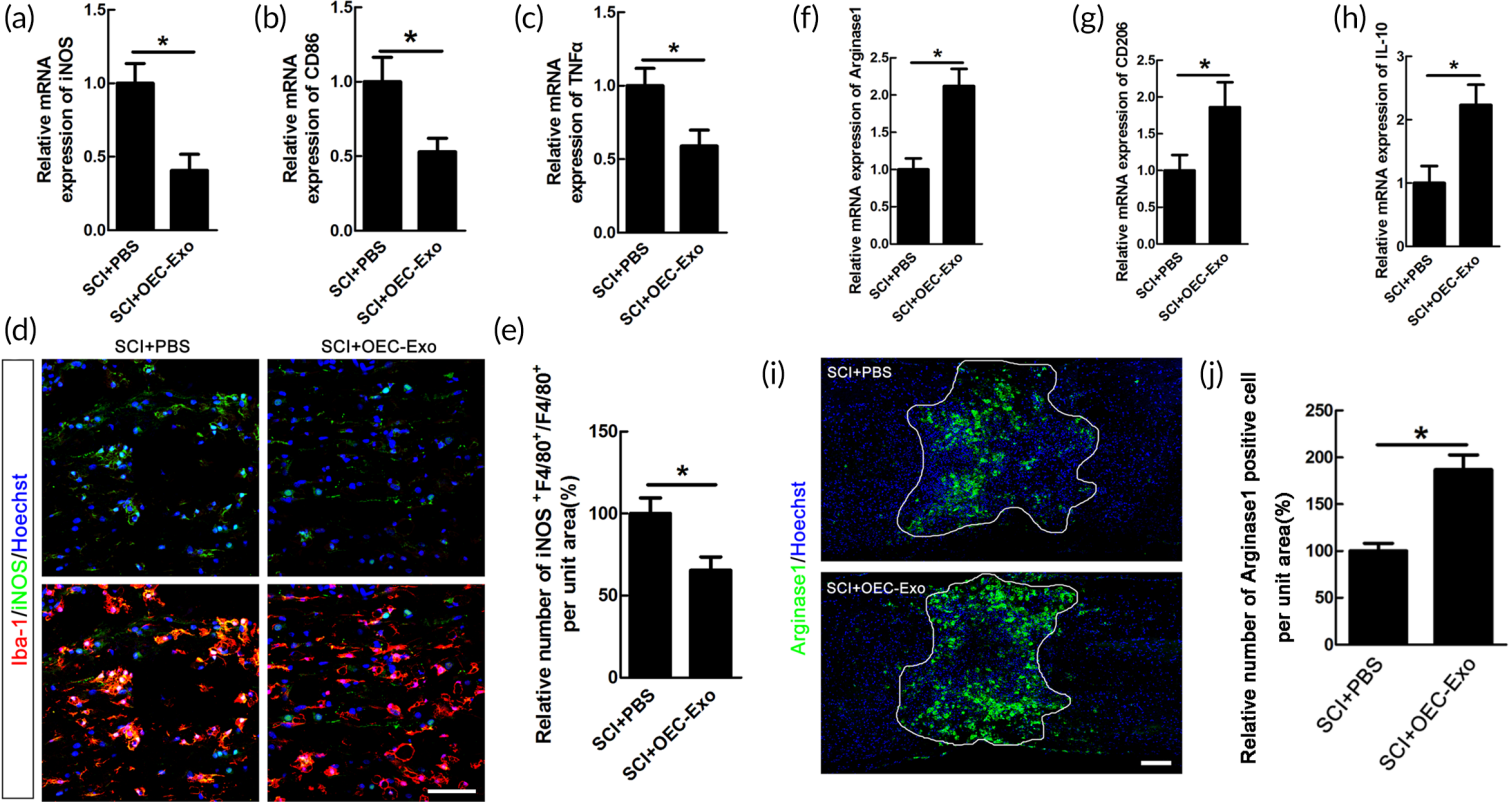
Efferocytosis of OECs‐Exo by microglia/macrophages alleviated their pro‐inflammatory polarization and increased the anti‐inflammatory cells after SCI. Quantification of pro‐inflammatory mRNA of iNOS (a), CD86 (b), and TNFα (c) in PBS or OECs‐Exo‐treated rats at 14 dpi. *N* = 6/group, *p < 0.05. (d) Representative images of staining of iNOS and Iba‐1 in PBS or OECs‐Exo‐treated rats at 14 dpi. Scale bar = 50 μm. (e) Quantification of iNOS‐positive microglia/macrophages in the bilateral areas 2000 μm rostral and caudal to the lesion site. Notice that OECs‐Exo treatment decreased the numbers of iNOS‐positive microglia/macrophages. *N* = 6/group, *p < 0.05. Expression of M2‐associated mRNA transcripts of Arginase1 (f), CD206 (g), IL‐10 (h) in PBS or OECs‐Exo ‐treated rats at 14dpi. *N* = 6, *p < 0.05 versus SCI+ PBS control. (i,j) Immunohistochemistry Arginase1‐positive cells in PBS or OECs‐Exo‐treated rats at 14 dpi and quantification of staining. Lesion border defined by GFAP immunoreactivity. *N* = 6/group, *p < 0.05 versus SCI+ saline control. Scale bar = 300 μm

Based on the above results, OEC‐Exo was injected in injured rats to determine the effects of OEC‐Exo on polarization of macrophage/microglia after SCI. To trace OEC‐Exo in the spinal cord, OEC‐Exo were labeled in red with DiI before injection, and about 50% of the OEC‐Exo were taken up by microglia/macrophages around the lesion at different time points after SCI (*n* = 3, Figure S3a,b). Similar to data in vitro, the mRNA levels of pro‐inflammatory markers of iNOS, CD86, and TNFα were significantly decreased in OEC‐Exo‐treated group (*n* = 6, *p < 0.05, Figure 5a–c). Further, immunohistochemistry showed that the number of iNOS‐positive macrophage/microglia was significantly decreased upon OEC‐Exo treatment after SCI (*n* = 6, *p < 0.05, Figure 5d,e). Meanwhile, we examined the effect of OEC‐Exo on anti‐inflammatory polarization. Our data of qPCR showed that OEC‐Exo significantly increased the mRNA levels of Arginase1, CD206, and IL‐10 (*n* = 6, *p < 0.05, Figure 5f–h). The result of immunostaining further revealed that the Arginase1‐positive macrophages/microglia in the epicenter was significantly increased in the OEC‐Exo‐treated group (*n* = 6, *p < 0.05, Figure 5i,j). These data suggested that OEC‐Exo treatment could partially inhibit the pro‐inflammatory polarization of macrophage/microglia and enhance its anti‐inflammatory polarization after SCI.

### 3.6 OEC‐Exo alleviated neuronal death and increased axon preservation after SCI

**FIGURE 6:**
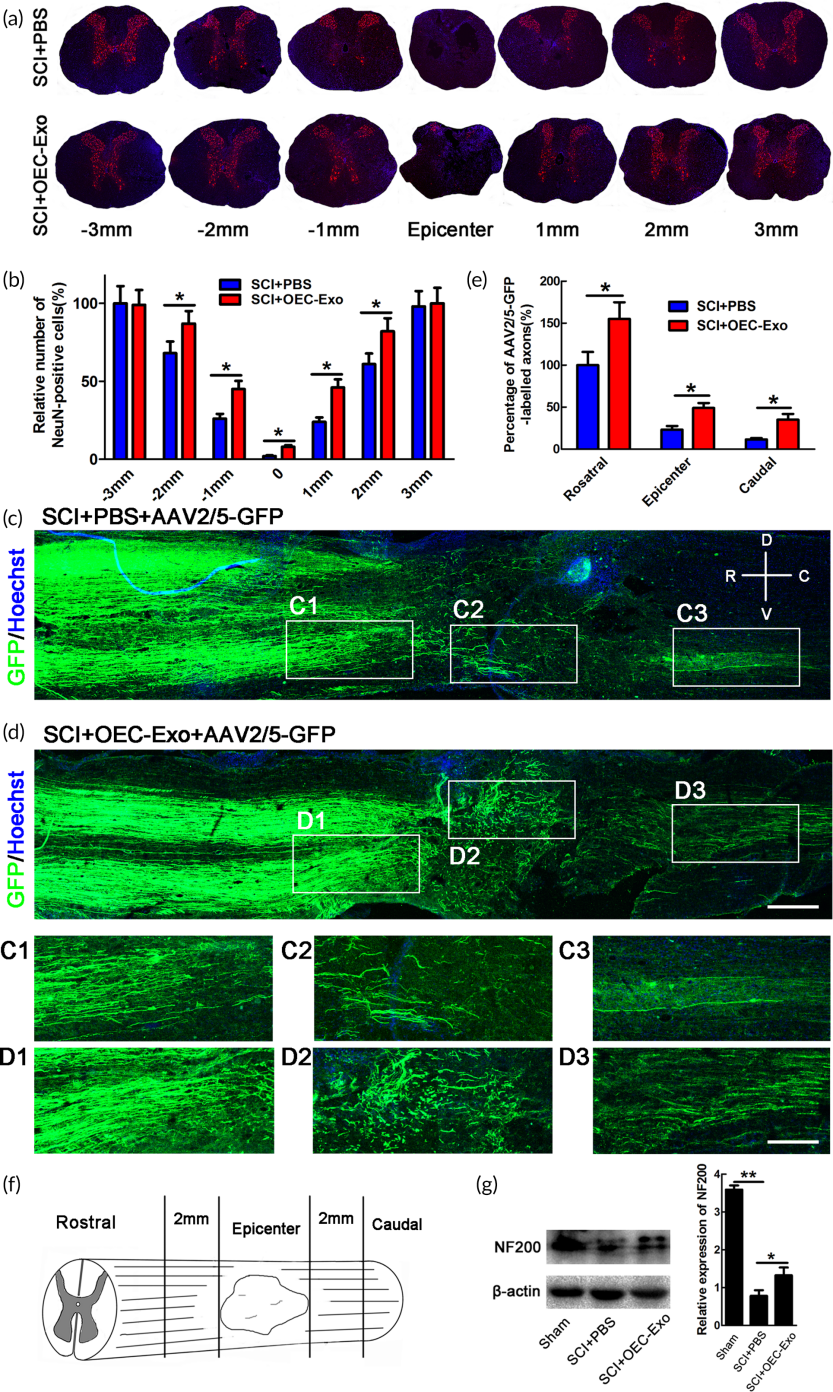
The effect of OECs‐Exo treatment on neuronal survival and preservation of axons after SCI. (a) Representative images of NeuN‐immunostaining in transverse sections in PBS‐ or OECs‐Exo‐treated rats at 4 wpi. The area of 3000, 2000, 1000 μm rostral and caudal to the lesion, as well as epicenter was selected to analyze. Scale bar = 300 μm. (b) Quantification of NeuN‐positive cells. Note that OECs‐Exo treatment significantly increased the numbers of NeuN‐stained neurons. *N* = 6/group, *p < 0.05. (c,d) Representative images of AAV2/5‐GFP‐labeled axons in longitudinal sections at 4 wpi in different groups. The high magnification images (C1–C3, D1–D3) were from the low magnification images in epicenter, rostral, and caudal areas of 2000 μm adjacent to lesion. Scale bar = 800 μm in low magnification images and 300 μm in high magnification images. Note that there are some axons present at caudal to the epicenter, demonstrating that our SCI model was incomplete. (e) Quantification of AAV2/5‐GFP‐labeled axons. *N* = 6/group, *p < 0.05. (f) The area on which quantification was performed. (g) Expression and quantification of NF200 in sham, SCI + PBS, or SCI + OECs‐Exo treatment. *N* = 3, *p < 0.05, **p < 0.01

Our previous study and other research works had demonstrated that pro‐inflammatory phenotype of microglia is closely related to neuronal death, and the latter, to a large extent, determines the severity of neurological disorders.^22^ NeuN‐immunostaining was performed to assess the effect of OEC‐Exo on neuronal survival. The data showed that the number of NeuN‐positive neurons in the area of 2000 μm, 1000 μm rostral and caudal to the lesion, as well as epicenter was significantly increased in OEC‐Exo ‐treated rats at 4 wpi (*n* = 6, *p < 0.05, Figure 6a,b).

AAV2/5‐GFP virus tracing was further performed to determine the effect of OEC‐Exo on axons preservation after SCI. The results showed that OEC‐Exo increased the AAV2/5‐GFP‐labeled axons in epicenter and area of 2000 μm away from each side of the lesion at 4wpi after SCI (*n* = 6, *p < 0.05, Figure 6c–f). We further confirmed this beneficial effects of OEC‐Exo by western blotting for NF200 (*n* = 3, **p < 0.01,*p < 0.05, Figure 6g). The above data indicated that OEC‐Exo treatment markedly increased the preservation of axons after SCI.

### 3.7 OEC‐Exo improved motor recovery after SCI

**FIGURE 7:**
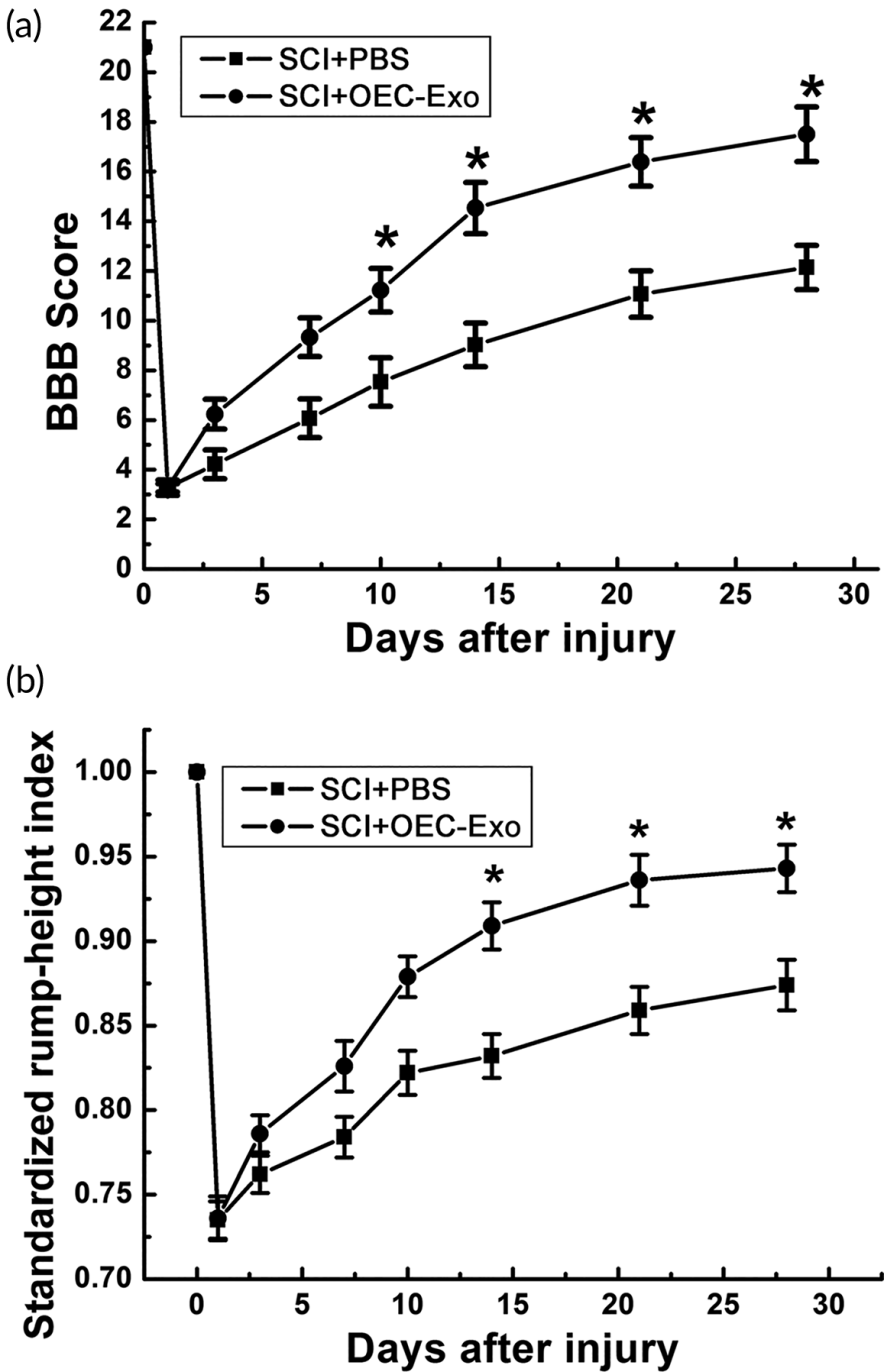
OECs‐Exo treatment facilitated functional recovery in rats. (a) BBB scores at different time points after SCI. (b) SRHI values at different time points after SCI. Note that higher BBB scores and SRHI values in OECs‐Exo‐treated rats. *N* = 6/group, *p < 0.05

To further investigate the effect of OEC‐Exo on locomotor recovery, hindlimb locomotor behavior was assessed by BBB locomotor rating scale and RHI assay before injury and at 1, 3, 7, 10, 14, 21, 28 days after SCI. The rats in OEC‐Exo group exhibited significant improvements in locomotor function as shown by higher BBB scores from 10 dpi and higher lift of the hind limbs from 14 dpi (*n* = 8, *p < 0.05, Figure 7a,b).

## 4 DISCUSSION

**FIGURE 8:**
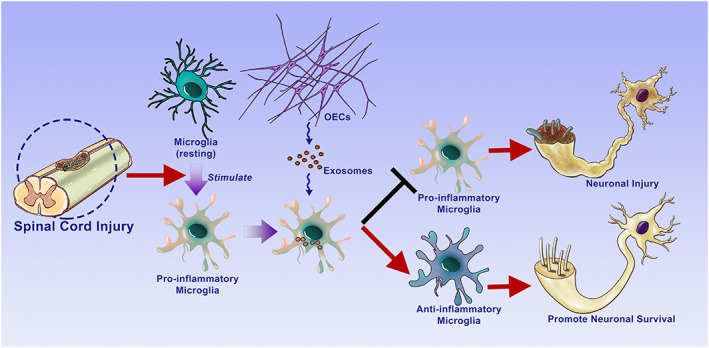
Schematic diagram showing the outline of our findings. OECs‐Exo provides neuroprotection for SCI by switching the phenotype of macrophages/microglia

In this study, we demonstrated that OECs‐Exo efficiently inhibited the pro‐inflammatory polarization of macrophage/microglia while enhanced the numbers of anti‐inflammatory cells. We further showed that OECs‐Exo promoted neuronal survival, increased preservation of axons, and facilitated functional recovery after SCI. Our study provides a valuable reference for immunomodulation by exosome and a promising strategy for the treatment of SCI (Figure 8).

Cell‐based therapy, regarded as one of the most promising approaches for SCI, has been studied for a long period.^4^, ^40^, ^41^ Although OECs were reported to have several beneficial effects including neuronal protection, axonal elongation, and immunomodulation, the inevitable problems such as low survival rates and immune rejection limited their clinical application.^8^, ^9^, ^42^, ^43^, ^44^, ^45^ In view that exosomes could play critical roles in intercellular communications, exosomes‐based therapies have been applied to SCI.^46^, ^47^ It is reported that hypoxic preconditioned mesenchymal stem cell (MSC) derived exosomes could repair traumatic SCI by shifting microglial polarization via miR‐216a‐5p.^48^ In addition, embellished MSC‐Exo with small interfering RNA (siRNA) to phosphatase and tensin homolog (PTEN) enhanced axonal growth and neovascularization, which significantly elicited functional recovery in rats with complete SCI.^49^ In this study, we found that OECs‐Exo modulated polarization of microglia/macrophage post injury and promoted functional recovery after SCI. Consistent with the other researches and our previous studies,^19^, ^50^ NF‐κB signaling pathway has been verified to participate in regulation of M1polarization of microglia in the present study. In addition, we also found that c‐Jun signaling was involved in OEC‐Exo mediated phenotypic shift of microglia. Although we demonstrated that the NF‐κB and c‐Jun signaling involved in phenotype switching of microglia/macrophage, it still remains unclear that what specific substances in OECs‐Exo mediated the upstream. It will be studied in our future research.

In this study, although we found that OECs‐Exo promoted the neuronal survival and axon preservation, the specific mechanism is still unsolved and is worthy to be studied in the future. First, although ameliorated immune microenvironment could support neuronal survival, about 5% of OEC‐Exo (data not shown) was found to be engulfed by neurons, which may contribute to the enhanced neuronal survival and axon preservation itself. Second, in our incomplete injury model, we could not exclude the possibility that the increased number of GFP‐labeled axons may also be due to the increased number of rescued neurons. In addition, in control group of our model, GFP‐labeled axons could be observed in the caudal of the epicenter, supporting that the crush was an incomplete injury model.

It has been reported that the activated microglia/macrophages were responsible for pathological changes including neuronal death, demyelination.^51^, ^52^, ^53^, ^54^, ^55^, ^56^, ^57^ We therefore focused on the OEC‐Exo‐microglia/macrophage interaction, and we found that OEC‐Exo could be internalized by microglia/macrophages after SCI. Based on the above data, we speculated that the neuronal protection of OECs‐Exo was primarily mediated by ameliorated microglia/macrophages‐mediated immune microenvironment. Nevertheless, all types of cells were theoretically considered to have phagocytic ability. As recent study demonstrated that Alpha B‐crystallin (CryAB) in OECs‐Exo ameliorates the growth‐inhibitory environment created by neurotoxic reactive astrocytes through blocking nuclear NF‐κB translocation, we do not exclude the possibility of other residual cell types mediated the neuroprotection of OEC‐Exo.^58^

For studying extracellular vesicles (EVs) uptake, the use of fluorescently or radioactively labeled particles is the most commonly used approach.^59^ In this study, fluorescent labeling was applied due to easier handling. However, the limitations of this method had been reported in several studies.^60^, ^61^ By using CellMask Orange fluorescent lipophilic membrane dye, Takov K reported that lipophilic dyes are not specific to EVs and false‐positive results could be observed due to the labeled lipoproteins.^60^ In this study, DiI dyes was applied and it might intercalate into undesirable extracted lipoprotein, we thus could not exclude the false positives even after extensively washing when labeling and fasting of the animals as recommended.^60^ Another important problem is that the labeled EVs could diffuse to other cellular membranes,^62^ leading to the misinterpretation of the results. It has been reported that PKH dyes exhibits an in vivo half‐life ranging from 5 to 100 days,^63^ which extremely exceeds the half‐life of EVs (up to 24 h in in vitro and from 30 min to 6 h in vivo).^62^, ^64^ In this study, lipophilic dyes could be observed at 5 d and 10 dpi, which might not reflect the real EVs half‐life circulation. Although novel labeling methods has been reported,^62^, ^64^, ^65^ technical challenge limits its widespread use. The development of specific and easily used dyes might help studying the uptake of exosomes.

Our data proved that exosomes may largely contribute to the neuroprotective properties of transplanted OECs in SCI, and will provide a novel direction for exosomes‐based therapy for SCI.

## AUTHOR CONTRIBUTIONS

**Hong Fan:** Conceptualization (equal); data curation (equal); formal analysis (equal); investigation (equal); methodology (equal); project administration (equal); writing – original draft (equal); writing – review and editing (equal). **Zhe Chen:** Data curation (equal); investigation (equal); methodology (equal); writing – original draft (equal). **Hai‐Bin Tang:** Data curation (equal); methodology (equal); project administration (equal). **Le‐Qun Shan:** Software (equal); validation (equal); visualization (equal). **Zi‐Yi Chen:** Resources (equal); software (equal); validation (equal). **Xiao‐Hui Wang:** Resources (equal); software (equal); visualization (equal). **Da‐Geng Huang:** Resources (equal); software (equal); visualization (equal). **Shi‐Chang Liu:** Resources (equal); software (equal); visualization (equal). **Xun Chen:** Resources (equal); software (equal). **Dingjun Hao:** Conceptualization (equal); data curation (equal); formal analysis (equal); funding acquisition (supporting); investigation (equal); methodology (equal); project administration (equal); resources (equal); software (equal); supervision (lead); validation (equal); visualization (equal); writing – original draft (equal); writing – review and editing (equal).

## CONFLICT OF INTERESTS

The authors declare that there are no conflict of interests.

## Supporting information


**Appendix** **S1**: Supporting Information

## Data Availability

The data used to support the findings of this study are available from the corresponding author upon request.
