# Supplementary material for: Exosomes derived from olfactory ensheathing cells provided neuroprotection for spinal cord injury by switching the phenotype of macrophages/microglia
Source: Bioeng Transl Med. 2021 Dec 28;7(2):e10287. doi: 10.1002/btm2.10287 (PMC9115713; doi:10.1002/btm2.10287)
Supplement: Supplementary file 1 — Appendix S1: Supporting Information [file BTM2-7-e10287-s001.pdf]

Supplementary materials

Supplementary Figure 1. Purity of primary cultured microglia. **(A)** Phase-contrast image of primary cultured microglia. **(B)** Purity of microglia was identified by Iba1 staining. Scale bar = 30 μm. **(C)** Quantification of the Iba1-positive microglia. Note that about 90% of the cells are Iba1 positive.

Supplementary Figure 2. Effects of OECs-Exo on expression of IRF3 and p-c-Jun upon LPS treatment. **(A)** The mRNA level of IRF3 in microglia under normal condition, LPS or LPS plus OECs-Exo treatment. **(B)** Quantification of expression level of p-c-Jun in microglia under control, LPS or LPS plus OECs-Exo treatment. N = 3, *P < 0.05, **P < 0.01. **(C)** Immunostaining of p-c-Jun and F4/80. Scale bar = 30 μm. **(D-E)** Quantification of the IFI/area and the nuclei/cytosol fluorescence intensity ratio of p-c-Jun. Note that OECs-Exo significantly supressed the increased IFI and nulear translocation of p-c-Jun induced by LPS. N = 3, *P < 0.05, **P < 0.01, ***P < 0.001.

Supplementary Figure 3. (A) Representative confocal microscopy images showing uptake of OECs-Exo (red) by microglia/macrophages (green) in spinal cord at 1dpi, 5dpi, 10dpi. Scale bar=50 μm. (B) Quantification of the percentage of internalization of OECs-Exo by microglia/macrophages. Results are presented as mean ± SEM. N = 3/group.

Supplementary Figure 4. Full gels of CD63, CD9, pNF-κB, NF200, p-c-Jun, β-actin and GAPDH.

Sup Table 1. PCR primer sequences

Gene	Forward primer (5'-3')	Reverse primer (5'-3')
iNOS	GCCTAGTCAACTACAAGCCCC	AGAAACTTCCAGGGGCAAGC
CD86	AGACATGTGTAACCTGCACCAT	ACTTTTTCCGGTCCTGCCAA
IL-12	GTGTCAATCACGCTACCTCCT	CTTGGCAGGTCCAGAGACTG
IL-18	CAGCTCTTCTACCAGCAAACAT	CTTCCAACTGAGAGGCTGTGC
CD206	GAGGACTGCGTGGTGATGAA	CATGCCGTTTCCAGCCTTTC
Arginase1	AAGATGTGCCCTCTGTCTTTTAG	CCCCTCCTCGAGGCTGTC
IL-4	CCTTGCTGTCACCCTGTTCT	CGGTGCATGGAGTCCCTTTT
IL-10	CGCTGTCATCGATTTCTCCC	TAGACACCTTTGTCTTGGAGCTTAT
TNFα	ATGGGCTCCCTCTCATCAGT	GCTTGGTGGTTTGCTACGAC
IRF-3	TGGCTGCGAGTCTCAACTAC	CAGTTCCTGAGCCAGGGGAG
GAPDH	GGCTCTCTGCTCCTCCCT	GTCTATGAGACGAGGCTGGC

Sup Figure 1

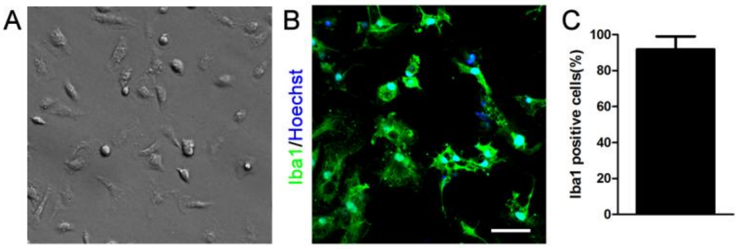

Sup Figure 2

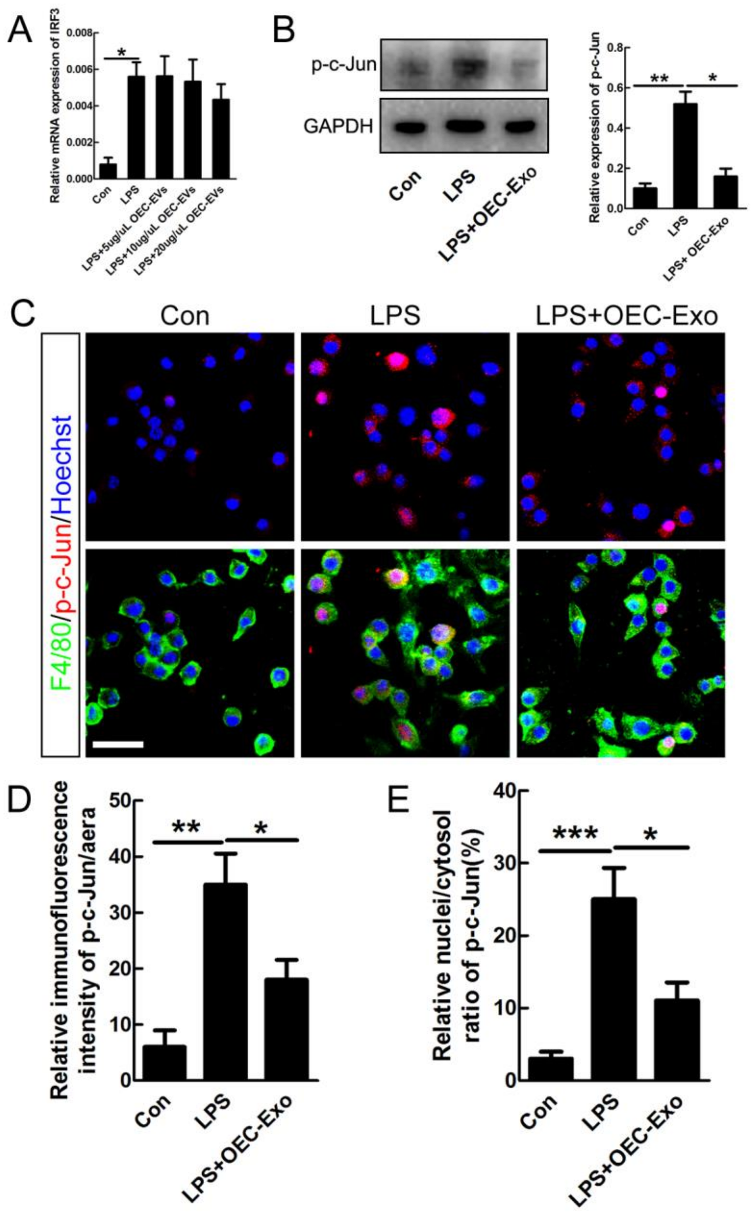

A
Relative mRNA expression of IRF3
0.008
0.006
0.004
0.002
0.000
*
Con
LPS
LPS+5ug/uL OEC-EVs
LPS+10ug/uL OEC-EVs
LPS+20ug/uL OEC-EVs
B
p-c-Jun
GAPDH
Con
LPS
LPS+OEC-Exo
Relative expression of p-c-Jun
0.8
0.6
0.4
0.2
0.0
**
*
Con
LPS
LPS+ OEC-Exo
C
Con
LPS
LPS+OEC-Exo
F4/80/p-c-Jun/Hoechst
D
Relative immunofluorescence intensity of p-c-Jun/aera
50
40
30
20
10
0
**
*
Con
LPS
LPS+OEC-Exo
E
Relative nuclei/cytosol ratio of p-c-Jun(%)
40
30
20
10
0
***
*
Con
LPS
LPS+OEC-Exo

Sup Figure 3

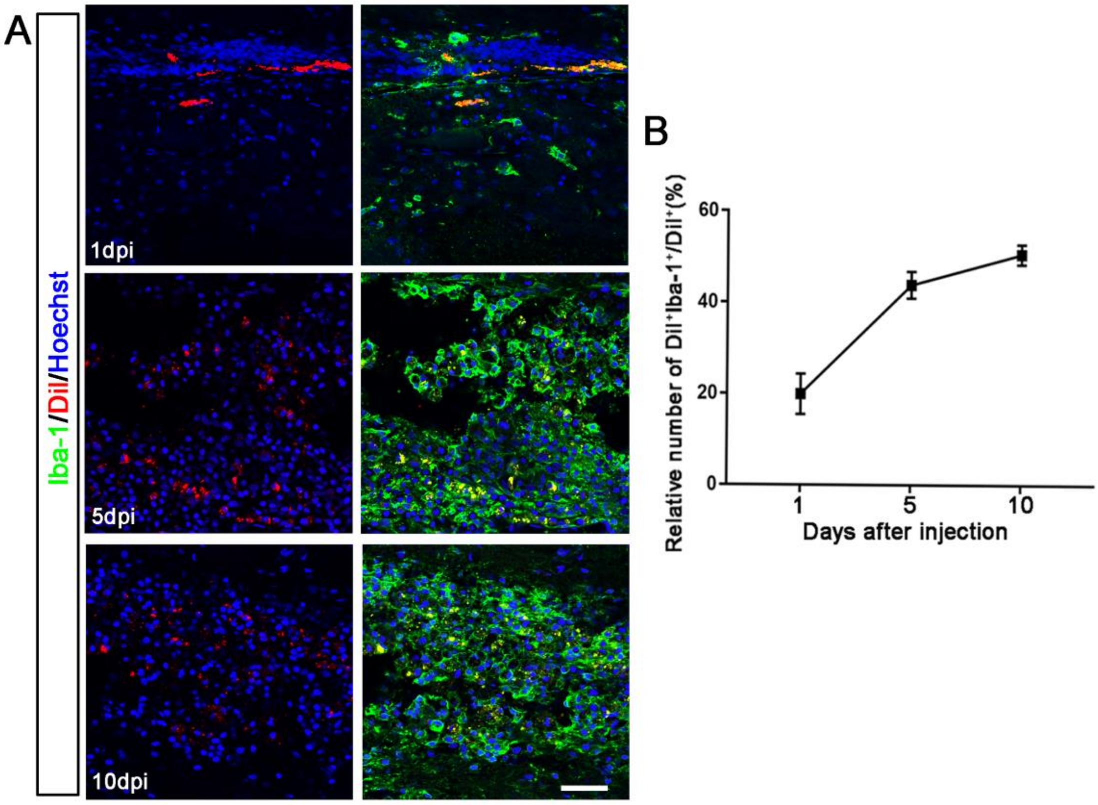
A
Iba-1/DiI/Hoechst
1dpi
5dpi
10dpi
B
Relative number of DiI⁺Iba-1⁺/DiI⁺(%)
60
40
20
0
1
5
10
Days after injection

Sup Figure 4

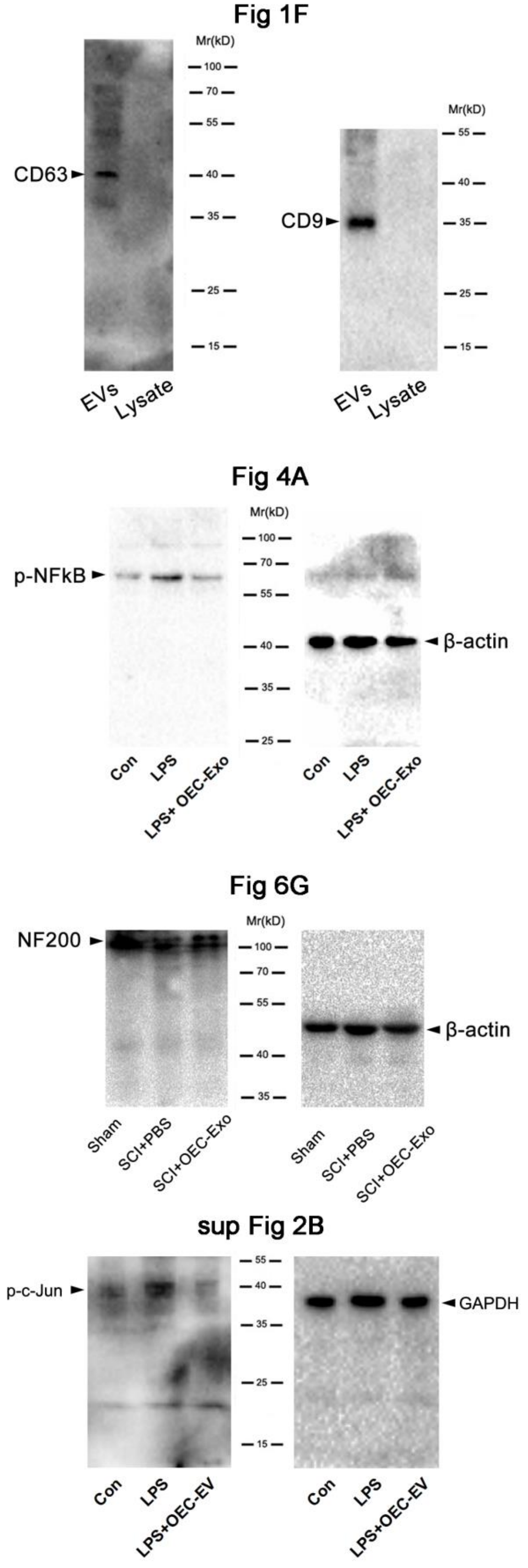
Fig 1F
Mr(kD)
100
70
55
40
35
25
15
CD63
EVs
Lysate
Mr(kD)
55
40
35
25
15
CD9
EVs
Lysate
Fig 4A
Mr(kD)
100
70
55
40
35
25
p-NFkB
β-actin
Con
LPS
LPS+ OEC-Exo
Con
LPS
LPS+ OEC-Exo
Fig 6G
Mr(kD)
100
70
55
40
35
NF200
β-actin
Sham
SCI+PBS
SCI+OEC-Exo
Sham
SCI+PBS
SCI+OEC-Exo
sup Fig 2B
55
40
35
25
15
p-c-Jun
GAPDH
Con
LPS
LPS+OEC-EV
Con
LPS
LPS+OEC-EV
